# Evaluation of the use of a Renal Health application by kidney transplant recipients

**DOI:** 10.1590/1518-8345.6039.3822

**Published:** 2023-01-30

**Authors:** Juliana Gomes Ramalho de Oliveira, Hélady Sanders-Pinheiro, Ronaldo Almeida de Freitas, José Eurico Vasconcelos, Marjan Askari, Geraldo Bezerra da Silva

**Affiliations:** 1 Universidade de Fortaleza, Fortaleza, CE, Brazil.; 2 Scholarship holder at the Fundação Cearense de Apoio ao Desenvolvimento Científico e Tecnológico (FUNCAP), Brazil.; 3 Universidade Federal de Juiz de Fora, Faculdade de Medicina, Juiz de Fora, MG, Brazil.; 4 Universidade de Fortaleza, Núcleo de Aplicação em Tecnologia da Informação, Fortaleza, CE, Brazil.; 5 Erasmus University, School of Health Policy & Management, Rotterdam, South Holland, Netherlands.; 6 Scholarship holder at the Conselho Nacional de Desenvolvimento Científico e Tecnológico (CNPq), Brazil.

**Keywords:** Nephrology Nursing, Health Strategies, Medication Adherence, Kidney Transplantation, Self-Care, Implementation Science, Enfermagem em Nefrologia, Estratégias de Saúde, Adesão à Medicação, Transplante de Rim, Autocuidado, Ciência da Implementação, Enfermería Nefrológica, Estrategias de Salud, Adherencia a la medicación, Trasplante de riñón, Autocuidado, Ciencia de la implementación.

## Abstract

**Objective:**

to evaluate the use of a renal health application by kidney transplant recipients.

**Method:**

a retrospective, observational study with a sample composed of individuals registered in the kidney transplant section of the application from July of 2018 to April of 2021. Demographic data, data entry, time of use, weight, blood pressure, blood glucose, creatinine, medication schedules, appointments, and tests were the variables collected. Descriptive analysis of the data was performed.

**Results:**

eight hundred and twenty-three downloads of the application were identified, and 12.3% of those were registered as kidney transplant recipients, the majority from southeastern Brazil (44.9%), 36±11 years old, and female (59.1%). Of the sample, 35.1% entered information such as creatinine (62%), weight (58.2%), and blood pressure (51.8%). Most used the application for one day (63.3%) and 13.9% for more than one hundred days. Those who used it for more than one day (36.7%) recorded weight (69%), medication intake (65.5%) and creatinine (62%), and scheduled appointments (69%).

**Conclusion:**

the kidney transplant recipient section of the Renal Health application generated interest in the young population, but showed low adherence throughout the assessed months. These results offer a relevant perspective on the implementation of mHealth technologies in kidney transplantation.

## Resumo

**Objetivo:**

avaliar o uso do aplicativo *Renal Health* por transplantados renais.

**Método:**

estudo observacional retrospectivo com amostra composta por usuários que realizaram cadastro na seção para transplantados renais do aplicativo de julho de 2018 a abril de 2021. Foram coletadas as seguintes variáveis: dados demográficos, inserção de dados, tempo de uso, registros de peso, pressão arterial, glicemia, creatinina, horários das medicações, consultas e exames. Realizou-se análise descritiva dos dados.

**Resultados:**

houve 1.823 downloads do aplicativo e 12,3% cadastraram-se na seção para transplantados renais, a maioria do Sudeste do Brasil (44,9%), com 36±11 anos e do sexo feminino (59,1%). Da amostra, 35,1% inseriram informações como creatinina (62%), peso (58,2%) e pressão arterial (51,8%). A maioria utilizou o aplicativo por um dia (63,3%) e 13,9% por mais de cem dias. Os que utilizaram por mais de um dia (36,7%), inseriram peso (69%), agendaram consultas (69%), medicações (65,5%) e creatinina (62%).

**Conclusão:**

a seção para transplantados renais do aplicativo *Renal Health* despertou interesse na população jovem, mas apresentou baixa adesão ao longo dos meses avaliados. Esses resultados oferecem perspectiva relevante na implementação de tecnologias *mHealth* no transplante renal.

## Resumen

**Objetivo:**

evaluar el uso de la aplicación *Renal Health* por parte de los receptores de trasplante renal.

**Método:**

estudio observacional retrospectivo con una muestra compuesta por usuarios que se registraron en la sección de trasplantados renales dentro de la aplicación desde julio de 2018 hasta abril de 2021. Se recolectaron las siguientes variables: datos demográficos, ingreso de datos, tiempo de uso, registros de peso, presión arterial, glucosa en sangre, creatinina, esquemas de medicación, consultas y exámenes. Se realizó un análisis descriptivo de los datos**.**

**Resultados:**

Ocurrieron 1.823 descargas de la aplicación y 12,3% se registró en la sección de trasplantados, la mayoría del sudeste de Brasil (44,9%), con edad de 36±11 años y del sexo femenino (59,1%). De la muestra, 35,1% ingresó información como: creatinina (62%), peso (58,2%) y presión arterial (51,8%). La mayoría utilizó la aplicación durante un día (63,3%) y el 13,9% más de cien días. Quienes lo usaron por más de un día (36,7%), agregaron peso (69%), programación de consultas (69%), medicación (65,5%) y creatinina (62%).

**Conclusión:**

la sección para trasplantados renales de la aplicación *Renal Health* despertó interés en la población joven, pero mostró baja adherencia en los meses evaluados. Estos resultados ofrecen una perspectiva relevante en la implementación de tecnologías *mHealth* en el trasplante renal.

**Highlights**

(1) Renal transplant recipients with interest in the renal health application were young.

(2) The data most commonly entered were weight, appointment scheduling, and taking medications.

(3) There was low adherence to use of the application without professional encouragement.

(4) Better dissemination and professional support can promote greater engagement

## Introduction

Electronic health (eHealth) includes digital tools and solutions that encompass Information and Communication Technology (ICT) services to support health^1^ are growing in popularity as access to the Internet increases^2^. Mobile health (mHealth) tools, an eHealth component focused on providing health services and information through mobile and wireless technologies^1^, have been prominent in the last decade. A massive 99.5% of Brazilian residences with Internet access used a smartphone for this purpose in 2019^2^. 

The scenario of a pandemic, with mobility restrictions and health care in isolated regions can potentiate the range of mHealth as a result of easy, timely, and personalized access to information needed for self-management^3^. The results of studies on the implementation of these digital strategies in different clinical settings demonstrate their applicability^4^ ^-^ ^6^, the importance of individual-centered development^5^ ^,^ ^7^, potential to improve treatment management, and the need for more robust evidence on the impact of the use of these strategies on outcomes^8^.

Kidney transplantation (KT) is the treatment of choice in the most advanced stage of chronic kidney disease (CKD)^9^. However, patients need periodic follow-up with a specialized team for better outcomes, as well as self-care actions such as management of multiple medications, tests, appointments, and self-monitoring of infection and symptoms of rejection^10^.

To understand instructions and communication from the health team, skills defined as Health Literacy (HL) are essential for self-management of the post-KT plan of care^11^. However, low HL is frequent among transplant candidates and recipients^12^. Non-adherence to treatment, especially to medication, is also considered high in KT^13^ ^-^ ^14^, and the reasons for this are complex and multifactorial, and therefore represent a challenge for the entire healthcare team^15^.

Some eHealth tools have been developed and tested as a strategy to expand HL, improve self-monitoring, and reduce non-adherence in KT^8^ ^,^ ^16^ ^-^ ^19^. Although outcomes about the effectiveness of these tools in transplantation are not unanimous, researchers highlight them as promising^9^ ^-^ ^10^ ^,^ ^17^ ^,^ ^20^.

The Renal Health application is a pioneering mHealth initiative in Brazil, with specific sections for the general population (without CKD diagnosis), hemodialysis patients, and kidney transplant recipients. Information about CKD prevention and treatment, as well as self-monitoring features, is available within it. 

The aim of this study was to evaluate the use of the Renal Health application by kidney transplant recipients.

## Method

### Type of study

This was a retrospective, observational study that analyzed the experience of using the Renal Health application, from download to utilization characteristics, of kidney transplant recipients.

### Data collection site

The study was conducted using the Renal Health smartphone application database. The first version of the application was released in 2018, in Portuguese, and was developed by a group of health researchers and the Information Technology Application Nucleus (Núcleo de Aplicação em Tecnologia da Informação - NATI) of the University of Fortaleza. The second version, released in 2019, is available for free on the online stores for Android (https://play.google.com/store/apps/details?id=br.unifor.renalhealth&hl=pt-BR) and *iOS* (https://apps.apple.com/br/app/renal-health/id1485397798) platforms, in Portuguese, Spanish, and English languages.

The application development approach was individual-centered interaction design^21^. Usability assessments were performed with patients, with content validation by nephrology specialists, yielding excellent results^22^.

**Figure 1:**
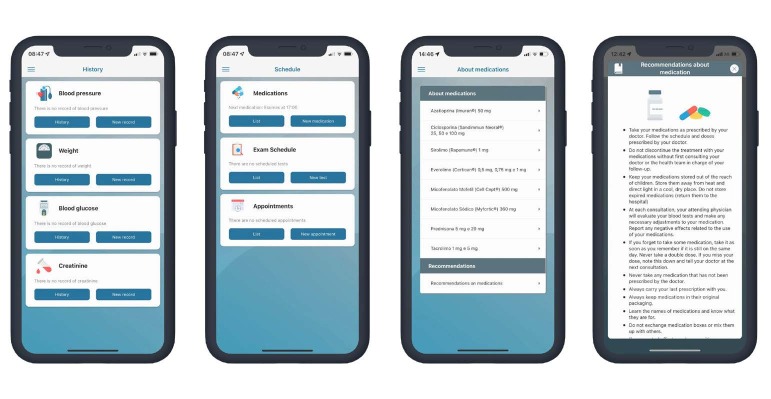
Screenshots from the kidney transplant section of the Renal Health application

Source: Renal Health application

In addition to information on various aspects of treatment, such as indications for and main side effects of immunosuppressants, signs and symptoms of infection and rejection, general and nutritional information, the section for kidney transplant recipients allows individuals to manually input data such as weight, blood pressure, blood glucose and creatinine levels, whose evolution can be graphically followed. The timetable for medications, appointments, and lab exams can be programmed, including an alarm option (Figure 1).

### Period

Individuals who downloaded the application in the period from July of 2018 to April of 2021 were analyzed. Data collection was performed in May of 2021.

### Population

The study population was composed of the 1,823 individuals who downloaded the Renal Health application in the period under review.

### Sample size

The sample consisted of the 225 individuals who had subscribed to the kidney transplant section during the study period.

### Study variables

The following demographic data were collected: age, sex, and Brazilian region of residence. For information about the ease and usability of the application, the following data were assessed: number of downloads, input of personal data, and time of use (number of accesses and period of use). The analyzed data related to self-monitoring were: weight, blood pressure, blood glucose, serum creatinine, medication schedule, alarm activation, scheduling of appointments and examinations.

### Data collection

Data were extracted from the PostgreSQL database, which hosts the content of the Renal Health application, available for researchers through the University of Fortaleza’s server. 

### Data treatment and analysis

Extraction, compilation, and descriptive analysis of the data were performed using the Microsoft Power BI (Business Intelligence) tool, desktop version (Redmond, Washington, USA). Continuous variables are presented as means and standard deviations, and categorical variables as percentages.

### Ethical aspects

The utilization of data from the Renal Health application for research was consented to by the individuals who agreed by signing the Terms of Free and Informed Consent form, sent after download, and approved by the Research Ethics Committee of the Universidade de Fortaleza (No. 4.134.607).

The treatment and analysis of the data entered into the application met the recommendations of Resolution 466/12 of the National Health Council and the General Law of Protection of Personal Data-LGPD.

## Results

The individuals registered in the kidney transplant recipient section of the Renal Health application were predominantly from the Southeast (44.9%) and Northeast (28.9%) regions of Brazil. The mean age was 36±11 years, and female sex prevailed (59.1%).

**Figure 2:**
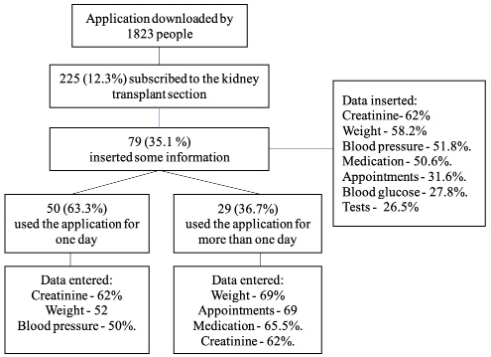
Characteristics of use of the renal transplant section of the Renal Health application from July, 2018 to April, 2021

Of the total number of registered individuals, 146 (64.9%) did not enter information into the application (Figure 2). 

Among those who entered data (35.1%), creatinine (62%), weight (58.2%), blood pressure (51.8%), medication intake scheduling (50.6%), appointments (31.6%), blood glucose (27.8%), and laboratory tests (26.5%) were the principle entries.

**Figure 3:**
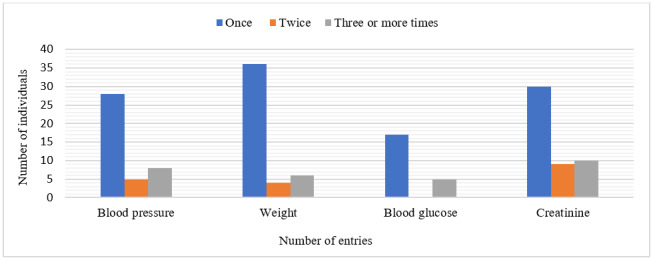
Number of individuals and self-monitoring data records in the kidney transplant section of the Renal Health application, from July of 2018 to April of 2021

With regard to the number of data inputs of the individuals who recorded their creatinine dosage results (62%), 30 did so only once (61.2%), nine twice (18.4%), and 10 three or more times (20.4%). Of the individuals who recorded weight data (58.2%), 36 did it only once (78.3%), four did so twice (8.7%), and six three or more times (13%). Among the individuals who recorded blood pressure data (51.8%), 28 did it only once (68.3%), five did so twice (12.2%), and eight entered it three or more times (19.5%). Blood glucose values were recorded by 27.8% of the individuals, of whom 17 did so only once (77.3%) and five did so three or more times (22.7%) (Figure 3).

Regarding scheduling for medications, 40 individuals scheduled their medications in their planners (50.6%), 35 immunosuppressants (87.5%), and 21 activated alerts (52.5%). 

Medical appointments were scheduled by 25 individuals (31.6%), 19 of them using the alert option (76%), and laboratory tests were scheduled by 21 (26.5%), 10 of which used the alert option (47.6%). The analysis of the period of time spent using the application showed that most individuals used it for only one day (63.3%), while 18.9% continued to use it for more than one month (Table 1).

**Table 1:** Period of kidney transplant recipient section use of the Renal Health application by individuals who entered information (n= 79), from July, 2018 to April, 2021. Fortaleza, CE, Brazil, 2021

Period of use	N (%)
1 day	50 (63.3)
2 - 31 days	14 (17.8)
32 - 100 days	4 (5.0)
>100 days	11 (13.9)

When analyzing the entered data of individuals who used the application for one day (63.3%), the most frequently explored sections were: creatinine level (62%), weight (52%), blood pressure (50%), and medication intake scheduling (42%).

Individuals who used it for more than one day (36.7%) recorded weight (69%), scheduled appointments (69%) and medications (65.5%), and creatinine level (62%) (Table 2).

**Table 2:** Functionalities and frequency of data entered by individuals using the kidney transplant recipient section of the Renal Health application for a period longer than one day (n= 29), from July/2018 to April/2021. Fortaleza, CE, Brazil, 2021

Functionalities used	N (%)	Entry frequency (mean±SD*)
Weight record	20 (69%)	3.0±3.5
Scheduling appointments	20 (69%)	1.1±0.3
Scheduling medication intake	19 (65.5%)	5.3±3.2
Creatinine level record	18 (62%)	2.7±2.8
Scheduling laboratory tests	15 (51.7%)	1±0
Blood pressure record	16 (55.1%)	3.1±3.4
Blood glucose level	9 (31%)	2.4±2.6

*SD = Standard deviation.

## Discussion

To the best of our knowledge, this is the first Brazilian study on the use of a treatment support application. The interest, according to the number of downloads and registration, and the use, in terms of frequency and preferences, of the section for kidney transplant recipients of the Renal Health app were analyzed. Although the application was created using an inclusive process^22^, i.e., the application was developed with the participation of patients and there was excellent acceptability^23^, a low frequency of use was found after the application was launched in online stores, which may be associated with dissemination of the application, the lack of encouragement by health professionals to use it, and the need for manual data entry.

Most participants were young women, living in populous regions of Brazil with a large number of kidney transplants^24^. The predominant age range in this study was lower than that of the Brazilian transplant population^13^ ^,^ ^25^, which may indicate a trend toward greater absorption of these technologies among young adults because of the greater ease in using smartphones and applications^26^. The percentage of Internet access in this age group is high, representing 90.4% in the Brazilian population^2^.

Individuals who subscribed to the section for kidney transplant recipients and did not include data (64.9%), limited themselves to health information and learning about the tool. In a study on the use of applications in diabetes management, this percentage was 57.3%, suggesting a behavior of superficial exploration of the applications or even a certain degree of insecurity about the methods of incorporating mHealth into the treatment regimen^27^. High initial acceptance rates and low actual use of the tools over time were also found among lung transplant recipients^4^.

Our findings showed that, among individuals who entered some data into the application, only 13.9% continued using it for more than 100 days. Low or declining engagement in eHealth applications has been reported previously in different groups, such as lung^5^, kidney^16^ and diabetes^27^ transplant recipients. Keeping the patient committed to these technologies throughout time is a challenge, and has motivated the development of several studies^28^ ^-^ ^31^. 

System data use is an important marker of engagement, as it signals what is engaging about an intervention. However, other measures are needed to assess the psychological aspects that influence perceptions, use, and effectiveness^32^. 

Among the self-monitoring functionalities, weight recording, scheduling appointments, medication intake, and creatinine level recording were the data with the highest percentages of entries by individuals who used the application for more than one day. This finding indicates the perception of transplant recipients about the importance of these aspects in the post-KT treatment. Adherence to immunosuppressive therapy is one of the most important factors in long-term graft survival^33^. It is known that blood pressure and blood glucose records may favor hypertensive and diabetic subgroups, and that engagement with specific modules of the tool is influenced by patient characteristics^27^.

Manual and continuous data entry is considered another decisive aspect in the low engagement of patients^27^, which may have influenced ongoing use in this study, and it is necessary to automate functionalities to overcome this barrier. Although we do not have more specific data available from our study, some factors have already been described as decisive in engagement with mHealth tools, such as personalization, communication, navigation, credibility, presentation, and tool layout^31^. Other important elements include self-monitoring, personalized feedback, gaming^34^, and the encouragement of use by professionals involved in care^26^ ^,^ ^30^. 

Newly transplanted patients may show greater acceptance of mHealth by contributing to the incorporation of care into the routine^16^. Therefore, it is likely that it is used only for initial learning and that there is a decline in its use over time. In this sense, additional studies are needed to better understand the reasons for low use and abandonment of mHealth tools, as in the case of Renal Health.

Previous research has shown that professional monitoring and encouragement of mHealth use can promote optimal patient engagement outcomes^26^, and planning is needed to integrate these technologies into the workflow of transplant programs^4^. However, even in interventions with eHealth tools guided by health professionals and researchers, such as in randomized clinical trials with a long follow-up period, it was found that encouraged habits are not sustained after the conclusion of the studies, due to the interruption of periodic reinforcement^4^. 

Three main challenges in mHealth design and implementation should be focused on: “maintaining adaptability and reducing complexity; maintaining positive beliefs about the intervention among those who deliver it, with goal setting, and providing feedback in a timely and understandable manner to key stakeholders”^35^. While prolonged use by the target audience is a relevant metric to qualify mHealth performance, the gold standard in assessing engagement and adherence to these tools is not yet well established^27^. Ongoing monitoring of mHealth outcomes is necessary, as it contributes to improvement and provides feedback for changes in implementation strategies^32^. 

Mobile health does not replace the health team-patient relationship, nor the traditional model of care, but it represents a tool capable of improving treatment effectiveness, considering the need for continuous supervision and professional encouragement to achieve sustained effects through its use^4^.

Regardless of the innovative nature of this study, some limitations need to be acknowledged. The sociodemographic and clinical data collected restrict the analysis of the sample reached by the application. In addition, although during the registration of these individuals, the inclusion of the health service in which they are being treated is requested, it is not possible to affirm that the individuals registered are exclusively kidney transplant recipients. The server that hosts the data does not provide information about patient access to the educational sections of the application, which limits the scope of the investigation on HL. In addition, the small number of individuals who entered data and used the application for a period longer than one day made it impossible to make an association between the variable. 

Future studies should be conducted to identify the reasons for low adherence to the application and determine whether encouraging use and incorporation into the post-KT care program of the Renal Health application could increase HL, encourage self-monitoring, and reduce non-adherence to treatment. Moreover, in order to make the application more attractive, other actions are planned for the next updates, such as automation of some features, gaming strategies, and the development of communication channels with the health team.

With the advance of ICT and the use of digital tools in several areas of people’s daily lives, it is necessary to develop strategies that integrate health care with this new technological routine. The gains in quality of care and clinical outcomes from the incorporation of these tools are under continuous investigation. The results presented so far are encouraging, because they indicate the potentialities and weaknesses in the implementation in different groups. As for kidney transplant recipients, interest in ICT tools for post-KT follow-up was demonstrated, although the findings indicate the need to discuss engagement strategies.

## Conclusion

The kidney transplant section of the Renal Health application sparked interest among the young population. The main functionalities used by the patients were for recording weight, scheduling appointments, medication intake, and creatinine level recording. However, the application showed low adherence throughout the months evaluated.

Although preliminary, the findings of this study offer a relevant perspective to be considered in the implementation of eHealth technologies in kidney transplant patients. A significant investment in the dissemination of the application as a support in post-KT care, in society, and in transplant centers, and the development of partnerships with health professionals who believe in the potential of this tool can provide more engagement of patients, with potential effects on outcomes.
